# Notes on Myotomy and Tenotomy

**Published:** 1841-10-09

**Authors:** Reynell Coates


### MEDICAL EXAMINER.

DEVOTED TO MEDICINE, SURGERY, AND THE COLLATERAL SCIENCES.

No. 41.] PHILADELPHIA, SATURDAY, OCTOBER 9, 1841. [Vol. IV.

### ORIGINAL COMMUNICATION.

### NOTES ON MYOTOMY AND TENOTOMY.

BY REYNELL COATES, M. D.

To the Editors of the Medical Examiner.

Gentlemen,—I regret extremely that unavoidable engagements rendered it impossible to communicate with your last number, and that the series of these notes is thus interrupted. As the like difficulty may occur again, I will endeavour to remedy the consequences as far as practicable by special references. After the little expose of the anatomical changes consequent upon severe cases of varus, given in my last, p. 616, (and the catalogue might have been extended much further; for nothing has been said, as yet, on the subject of the alterations observable in the knee, the hip, and the spine,) it must be evident that the simple division of the tendo Achilles is incapable of affecting, *directly*, any material alleviation of most of the causes of deformity existing in that form of club-foot. The only important result of the operation consists in giving the foot the power of flexion to any extent not interdicted by the modifications undergone by the ankle joint, and in a diminution of the force with which the os calcis is drawn inwards. After the section, the foot may undoubtedly assume, somewhat more readily, a position at a right angle with the leg; and one of the principal obstacles opposing the return of the os calcis to its proper relations with the astragalus is thus removed. But no effect is *or can be* produced upon the new formed ligaments between the misplaced tarsal bones which tend to perpetuate the deformity, nor upon the contracted plantar fascia and muscles, which,—with or without the aid of the adductors,—effectually prevent, in the first instance, any amendment of the malposition of the anterior upon the posterior portion of the foot.

Independently of the mechanical after-treatment, then, nearly all that is effected by the section of the tendon of the triple extensor, whether the adductors share the same fate or not, is the bringing down of the heel, and the reduction of so much of inward rotation of the foot as the new connections of the os calcis, together with the altered form of this bone and the astragalus, permit.

But the change in the form of the ankle joint, described in my last note, prevents a return of the foot to a rectangular position, except at the expense of a sub-luxation of the astragalus forward;—a result which has been observed in every case that I have yet seen, and one upon which depends the inability to flex the foot beyond the right angle, which is so constantly noticed by the most cautious reporters of cases.

Now, let us bear in mind the fact that the tenotomists do not very often divide any other tendon than the tendo Achilles—that they profess to have cured many of the worst cases of varus after this operation alone—that though they sometimes also perform the section of the tendon of the tibialis anticus, and even that of the flexor communis, yet they very rarely meddle with the fascia plantaris, or the shorter muscles of the sole of the foot, upon which the angular deformity and much of the rotation of the anterior half of the tarsus obviously depend.

In examining the reported results, it will be found that the operators claim to have brought the foot nearly or exactly into its proper position in a very short time—say from ten days to six months. And this has been accomplished seemingly with about equal facility, whether few or many tendons are divided; except that those who cut the fascia plantaris have had less difficulty in flattening the foot, as might be naturally expected.

Now, when the fascia is not touched, upon what depends the facility with which the foot is brought to the ground in an attitude to be useful? Certainly not upon the division of one or more of the tendons abovementioned; for, with the exception of the flexor communis, none of these can produce any material effect on the deformity in the middle of the tarsus; and the influence of the long flexor itself is trivial. All that is accomplished by the sections is the power of bringing down the heel by partially sub-luxating the astragalus, and that of abducting imperfectly the posterior half of the tarsus.

View these results independently of the after-treatment, and what will be the position of the anterior half of the foot in a complete case of varus? By reference to the rude outline of the relation of parts given in my last, (*p.* 617, *second column*,) it will be seen that, after the flexion and abduction, the toes must point downwards and inwards, instead of being directed exactly towards the opposite limb; and that the support of the body, if support it can be called, depends upon *the point of the little toe*, instead of the side of the metatarsal bone of that toe, as formerly.

If the flexor communis has been divided, perhaps *something* may be gained in a slight increase of flexibility at the articulations of the ossa calcis and astragalus with the anterior tarsal bones; but when the tendo Achilles alone is cut, no relief whatever is given to the angular deformity by the operation. It is evident, then, that the operations usually performed for club-foot of this variety, instead of effecting a cure, tend to place the foot in an attitude more useless than before, and one that must inevitably reproduce the former malposition very soon after the union of the tendons and the attempt to use the limb.

But the operators will protest, in some surprise no doubt, that such is not the position of any of the feet on which they have operated! And why is it not so? Simply because, immediately or shortly after the tenotomy, they resort to *mechanical treatment.* Though the heel may have "come down two, three, or four inches" directly, yet the changes in the form of the tarsal bone, with their articulations, the formation of new ligamentous connections between them, the shortening of the long calcaneo-cuboid and metatarsal ligament, as well as the contraction of the short muscles and the fascia plantaris, all render it necessary to employ mechanical forces to remove the deformity of which I speak; and it is as fortunate as it is wonderful, that these various contractions yield to moderate and well ordered mechanical extension with remarkable rapidity. But the great question is, would they not have yielded just as readily to the same means without the division of the tendons?

It is unnecessary to refer again to the occasional sections of other tendons than that of Achilles, in order to argue this point; for the cases represented as effectually cured after this operation alone, are quite as happy and successful as those subjected to the more extensive cutting; which, indeed, is now going out of favor even with many of its former advocates. But it is perfectly obvious that the tendo Achilles has nothing whatever to do with the deranged angular relations of the anterior and posterior part of the foot, either before or after the operation. It can neither cause them, nor maintain them; and the real causes of resistance to the return of the anterior portion to its proper relation with all the tarsal bones except the astragalus,—causes which have been sufficiently discussed—act just as forcibly after the operation as they do before. They can be overcome by just as little force in the one case as in the other; and until they are overcome, the mere flexion and abduction of the deformed foot, resulting from the tenotomy, is even worse than useless. Let us, then, consider what are the forces remaining unchecked after the operation, as usually performed, before the foot is rendered capable of available service.

The whole history of luxations teaches us, that the short muscles may readily undergo extensive modification in length, without permanently losing their functional powers. Muscles much shorter than those interested in the operation of tenotomy in cases of club-foot are stretched, to even greater extent than the latter are contracted, in every old case of dislocation of the humerus downwards and inwards; yet we do not find their functions destroyed by this change of relation. I have in my mind, at present, a case of such luxation, occurring within a year of the present time, in which the motions of the limb are already so far recovered as to render it altogether improper to correct the consequences of the original error of diagnosis on the part of the surgeon, by attempting to break up the new ligamentous adhesions between the head of the humerus and the new forming joint. But, though the muscles yield thus readily to extension *properly regulated*, the ligaments are much less tractable. The latter organs being endowed with much less active vital powers, demand proportionally more *time* for any important alteration of structure: yet it does not follow that they require more *force* for this purpose. Every physiologist well knows that the *pseudo-gracefulness* of an Elssler, (let the Athenian reader correct the word by the aid of his lexicon, if he think it illegitimate!) is due to a deformity of the articulations and their ligaments—yet the force required to produce this popular weakness is by no means considerable. It only demands *time and habit.*

But, when we propose to elongate parts by mechanical forces, the muscles will ever be found to yield much more rapidly than the ligaments. No one will deny this position so far as the tonic contraction or contractility of muscles is concerned—but the muscles will actually *increase in length under extension, by a positive modification of nutrition*, much more readily than the ligaments.

Now, the causes which prevent the removal of the angular deformity at the middle of the tarsus in varus, are dependent, for the most part, on ligamentous contraction; (for the fascia plantaris is as much a ligament in structure and function, as is the calcaneo-cuboid and metatarsal ligament,) while those which oppose the flexion and abduction of the foot in general, are mainly muscular, *and the muscles interested are long in proportion to their bulk.*

It follows, then, if my predicates be unexceptionable, that more mechanical force is requisite in removing the deformity of the middle joints of the tarsus in varus than in conquering the contraction of the extensors and adductors of the foot; or, in other words, that, after the usual operation of tenotomy, more mechanical force is still requisite than would be sufficient, if proper time were allowed, to render the operation unnecessary.

The reasoning on this last conclusion, however, omits one important predicate, which will be discussed hereafter—namely: the peculiar conditions of the contracted muscles in club-foot. With a view to simplicity in the argument,

I waive this predicate at present. How far it may modify our conclusions, will be seen in another place.

If, then, the mechanical forces required for rendering the foot useful after tenotomy be as great as those required to remove the most intractable portion of the deformity—namely: the angular derangement of the middle of the tarsus—without division of the tendons, what truth is there in the argument of the tenotomists against the purely mechanical treatment? They complain that in the latter method, the pain given to the patient is intolerable!—Why intolerable? There can be but one cause for this pain and the other inconveniences said to be experienced, viz: the excessive force required to accomplish the purpose. But, *if the force be rightly directed*, it need not be greater in this mode of treatment than in that preceded by division of the tendons. After that division, the same parts precisely require elongation, and to exactly the same extent. The varus can be converted into simple pes equinus, without the knife, by one who knows how to adapt machinery to a purpose, at least as readily as the anterior portion of the foot can be rendered flat after the division of tendons.—If, then, there be ulceration and excessive pain occasioned in the one process and not in the other, *the fault lies in the mechanist and not in the necessity of the case.*

Still; confining our attention, for the present, to the condition of the tarsus, to the exclusion of the mere flexion of the whole foot; allow me to ask what is gained by the division of the tendons? If any thing be gained, it must be merely a more favourable position of the anterior half of the foot—one in which we can apply mechanical forces with more facility and to better advantage. And how stands the question in relation to this matter?

While the tendons retain their integrity, the os calcis remains in a great degree fixed in its extended position for a considerable time, under any legitimate force: and this very circumstance greatly facilitates the application of forces to the anterior part of the foot, in order to compel it to undergo, in proper order, the reverse of the several motions by which the deformity has been brought to its height. The last of the series of changes of position, in perfect varus, is that by which the cuboid and scaphoid bones, already placed perpendicularly—the latter above the former—are drawn backward, so as to rest against the inner side of the ossa calcis and astragalus. Now, the correction of this misplacement requires that the anterior part of the foot should be drawn forward to a certain extent, until the two former bones are brought more nearly into relation with the original articular surfaces of the two latter. A force of this character can be much more readily applied when the foot is extended than when it is flexed—and so far as this portion of the deformity is concerned, the retraction of the tendo Achilles which is rendered nugatory by tenotomy, is a positive mechanical advantage.

Supposing the anterior part of the foot to be drawn forward sufficiently to prevent it from being interlocked against the ossa calcis and astragalus, (see page 617, second column,) what is the next indication? This part of the foot in still placed on its side, with the end of the metatarsal bone of the little toe on the soil; the sole directed obliquely backward and inward, and the toes pointing forward and inward. A single glance at the tarsus will show that the next indication is to cause the anterior part of the foot to rotate upon the posterior portion, for such are the forms of the astragalo-scaphoid, and the cuboido-calcanean articulations, that this simple rotation tends to bring the toes forward, and to remove their disposition to point inward. In other words, it reduces the foot to the attitude of simple but forcible adduction, rendering the whole sole as flat as the altered form of the tarsal bones will permit, while it also considerably diminishes the prominence of the anterior part of the astragalus on the instep.

In order to effect this rotation, our mechanical forces must be applied in such a manner as to twist the anterior portion of the foot outward, and this is precisely the most difficult motion to effect; yet it is absolutely necessary, if we wish to obtain a cure unattended by the exaggeration of the arch of the instep, which is so constantly observed in the quasi cures on record. The difficulty of this part of the process depends mainly on the disposition of the twisting force to disorder that part of every apparatus for club-foot which is adjusted to the leg; and, in the nature of things, it can never be properly effected without extending the apparatus to the thigh, and keeping the knee flexed during its action.—A necessity which has been strangely overlooked by most surgeons, though it formerly led to the construction of numerous complex *chairs* for club-foot, all of which compel the patient to undergo unnecessary and injurious confinement during the treatment.

Now, this requisite twisting is much more readily effected with the foot in a state of forced extension, than when it is in a flexed position; and *here also the rigidity of the tendo Achilles is a positive mechanical advantage*; for, after the division and elongation of the tendon, the tortion must be effected at the end of a much longer lever; and this very seriously enhances the difficulty.

The rotation just mentioned, is opposed, to a certain extent, by the permanent retraction of the long flexor of the toes, and somewhat more decidedly by the tibiales anticus and posticus, especially the latter, which is inserted into the tuberosity of the os scaphoides. That it is ever *necessary* to divide these tendons in order to effect the rotation, I have seen no reason to believe; but, if necessary, it is not during the *first*, but after some progress is made in the *second stage* of the mechanical treatment that the measure becomes requisite. Detmold, who has recently performed such incisions repeatedly, though a decided advocate for cutting the tendo Achilles in most cases, expressly states that he has not observed any decided advantage to follow the division of the tibialis anticus, and the posticus has been very rarely touched, though it is decidedly the more embarrassing muscle, from its direct opposition to the rotation of the scaphoid bone.

At the termination of the second stage of the mechanical treatment, (still leaving the degree of flexion of the whole foot out of view,) we should have the sole of the foot nearly as flat as in health; the relation between the os cuboides and the os calcis also nearly normal; the under surface of the little toe coming habitually to the soil; and the whole foot but little more adducted than is usual during the sleep of infancy. Still, however, the scaphoid bone would be partially rotated inward, and the os calcis would retain its adduction on the astragalus to a certain extent; so that the whole sole would present somewhat inward, and the unnatural prominence of the anterior end of the astragalus on the instep—though much diminished—would still continue.

I regret extremely that due regard to the proper allotment of space to the surgical department of your Journal, obliges me to close here for the present. The argument will be regularly continued in my next.

